# Physical exercise for brain plasticity promotion an overview of the underlying oscillatory mechanism

**DOI:** 10.3389/fnins.2024.1440975

**Published:** 2024-08-08

**Authors:** Xueyang Li, Xuehong Qu, Kaixuan Shi, Yichen Yang, Jizhe Sun

**Affiliations:** Physical Education Department, China University of Geosciences Beijing, Beijing, China

**Keywords:** physical exercise, brain waves, cortical oscillations, plasticity, EEG

## Abstract

The global recognition of the importance of physical exercise (PE) for human health has resulted in increased research on its effects on cortical activity. Neural oscillations, which are prominent features of brain activity, serve as crucial indicators for studying the effects of PE on brain function. Existing studies support the idea that PE modifies various types of neural oscillations. While EEG-related literature in exercise science exists, a comprehensive review of the effects of exercise specifically in healthy populations has not yet been conducted. Given the demonstrated influence of exercise on neural plasticity, particularly cortical oscillatory activity, it is imperative to consolidate research on this phenomenon. Therefore, this review aims to summarize numerous PE studies on neuromodulatory mechanisms in the brain over the past decade, covering (1) effects of resistance and aerobic training on brain health via neural oscillations; (2) how mind-body exercise affects human neural activity and cognitive functioning; (3) age-Related effects of PE on brain health and neurodegenerative disease rehabilitation via neural oscillation mechanisms; and (4) conclusion and future direction. In conclusion, the effect of PE on cortical activity is a multifaceted process, and this review seeks to comprehensively examine and summarize existing studies' understanding of how PE regulates neural activity in the brain, providing a more scientific theoretical foundation for the development of personalized PE programs and further research.

## 1 Introduction

**Figure 1:**
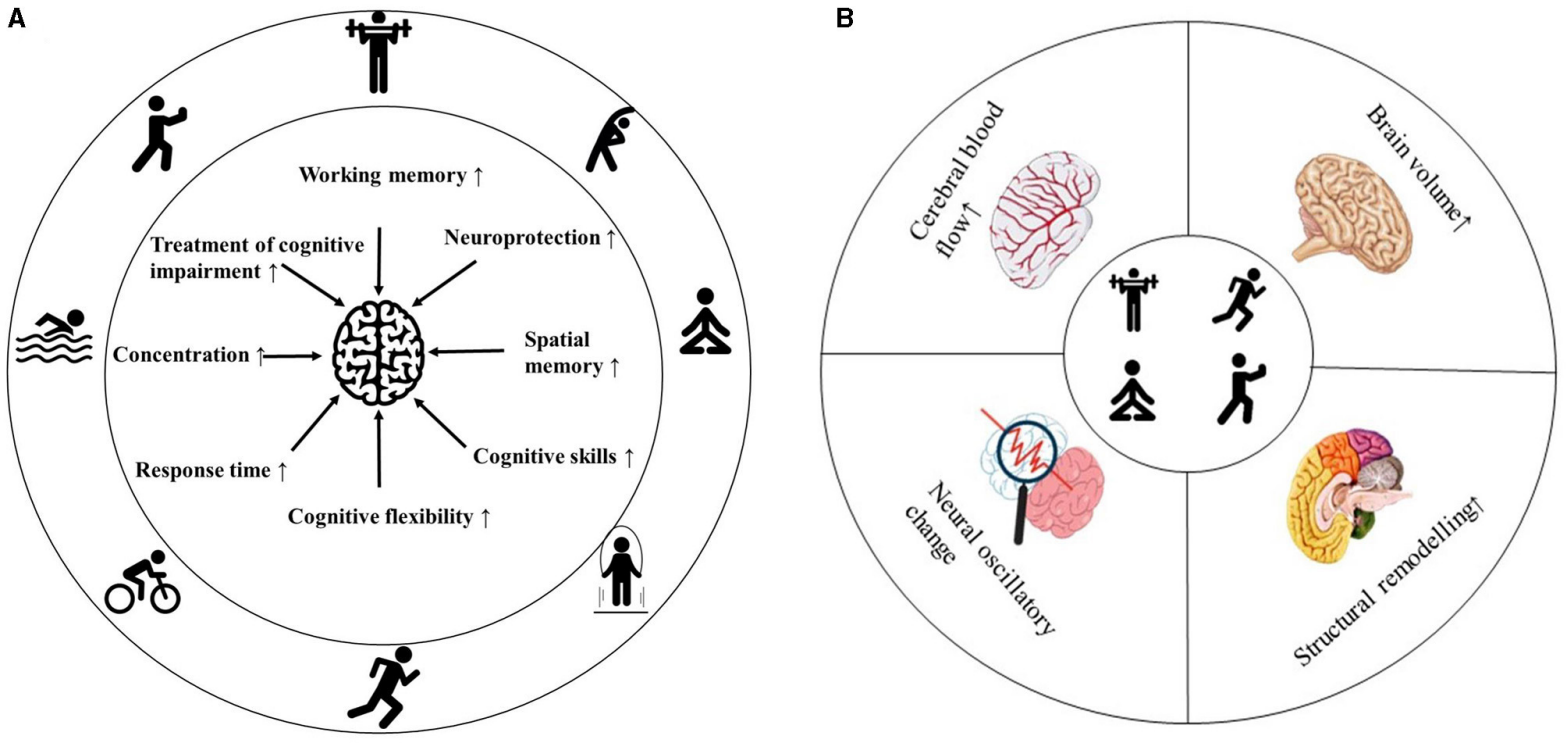
Summary of the effects of exercise on brain health. Effects of exercise on cognitive behavior **(A)**. Exercise-induced changes in brain physiology **(B)**.

The brain's plasticity refers to its ability to alter its structure and function, which is fundamental for learning, memory, and cognitive processing (Zhao et al., 2020). Physical exercise (PE) enhances neuronal activity and connectivity, thereby promoting brain plasticity through the modulation of neural networks and the facilitation of information transfer (Augusto-Oliveira et al., 2023). PE has emerged as a key modulator of brain plasticity, providing a promising strategy for mitigating cognitive decline and related diseases (Figure 1) (Voss et al., 2013). Neuronal oscillations, defined as rhythmic fluctuations in the electrical potential of groups of neurons, play a crucial role in integrating information within a common network (Rosenblum et al., 2022). These oscillations are essential drivers of interaction, communication, and information transfer in the brain and may be associated with certain mental disorders (Han, 2023; Wang et al., 2024). Non-invasive imaging techniques are significant methodologies for monitoring neural oscillations, with electroencephalography (EEG) being a commonly used tool in this field (Hosang et al., 2022). Various EEG frequency bands (delta, theta, alpha, beta, and gamma) are generated by distinct neuronal populations in different brain regions (Han et al., 2021a,b). Understanding the role of oscillations in neuronal function regulation is crucial for deciphering the effects of PE on the brain (Wang et al., 2023). Measuring neural oscillations before and after exercise can elucidate how physical activity influences cognitive function.

Physical activity (PA) encompasses any bodily movement that results in energy expenditure (EE) through skeletal muscle contraction. Physical exercise (PE), a key element of PA, is characterized by planned, structured, and repetitive physical movements aimed at improving or maintaining various components of physical fitness (Caspersen et al., 1985; Murphy et al., 2016). It includes activities such as brisk walking, running, swimming, cycling, ball games, dancing, and weight lifting (Qiu et al., 2023). Both PA and PE enhance cognitive performance by stimulating molecular mechanisms such as brain-derived neurotrophic factor (BDNF) (Vedovelli et al., 2017), learning (Guo et al., 2020), and memory (Wheeler et al., 2020). Interventions focused on increasing planned and organized activities often use the term “physical exercise” instead of “physical activity” (Gallardo-Gómez et al., 2022). To improve health, the American College of Sports Medicine recommends engaging in aerobic and resistance exercises on a regular weekly basis (Weiss et al., 2023). Studies find that PE significantly influences spatial memory, working memory, and executive attention (Chaire et al., 2020). Aerobic exercise enhances spatial memory by promoting hippocampal neurogenesis and increasing BDNF levels (El-Sayes et al., 2019; Stern et al., 2019). Resistance exercise, on the other hand, strengthens executive function by increasing prefrontal cortex volume and thickness (Chow et al., 2021). Research indicates that exercise intensity plays a crucial role in cognitive benefits, with high-intensity exercise potentially offering greater advantages than low-intensity exercise (Stern et al., 2019).

Moreover, various forms of mind-body exercises, such as yoga, Tai Chi, and dance, have gained popularity in recent years. Mind-body exercise employs a mind-body approach to achieve both physical and mental benefits through physical activity (Chan et al., 2017). Additionally, specific interventions like Positive Thinking Yoga have been shown to positively impact the mental wellbeing of individuals with Parkinson's disease (Kwok et al., 2019). Ultimately, the effectiveness of exercise in preventing or treating diseases is influenced by factors such as the type, duration, frequency, and intensity of the exercise (Guo et al., 2020; Qiu et al., 2023).

Thus, this review explores the possible neural mechanisms by which PA influences brain and cognitive functions by examining and synthesizing the relevant literature to date from four perspectives: (1) effects of resistance and aerobic training on brain health via neural oscillations; (2) how mind-body exercise affects human neural activity and cognitive functioning; (3) age-Related effects of PE on brain health and neurodegenerative disease rehabilitation via neural oscillation mechanisms; and (4) conclusion and future direction.

## 2 Effects of resistance and aerobic training on brain health via neural oscillations

Regular exercise at moderate intensity, encompassing activities such as strength training, endurance exercises, balance routines, flexibility exercises, and coordination drills, positively contributes to all aspects of human health (Qiu et al., 2023). However, sudden high-intensity workouts in untrained individuals can trigger adverse cardiovascular events (López-Otín and Kroemer, 2021). Hence, the intensity and nature of training play pivotal roles in producing beneficial health outcomes (Qiu et al., 2023). The protective impact of both short-term and long-term exercise on the central nervous system against neurodegeneration and cerebrovascular diseases has garnered significant interest from researchers (Liu et al., 2019; Qiu et al., 2023). Neuroplasticity is central to this Vints et al. (2022). Neuroplasticity, the brain's ability to undergo functional and structural changes in response to internal or external stimuli, plays a crucial role in this context (Voss et al., 2017b).

**Figure 2:**
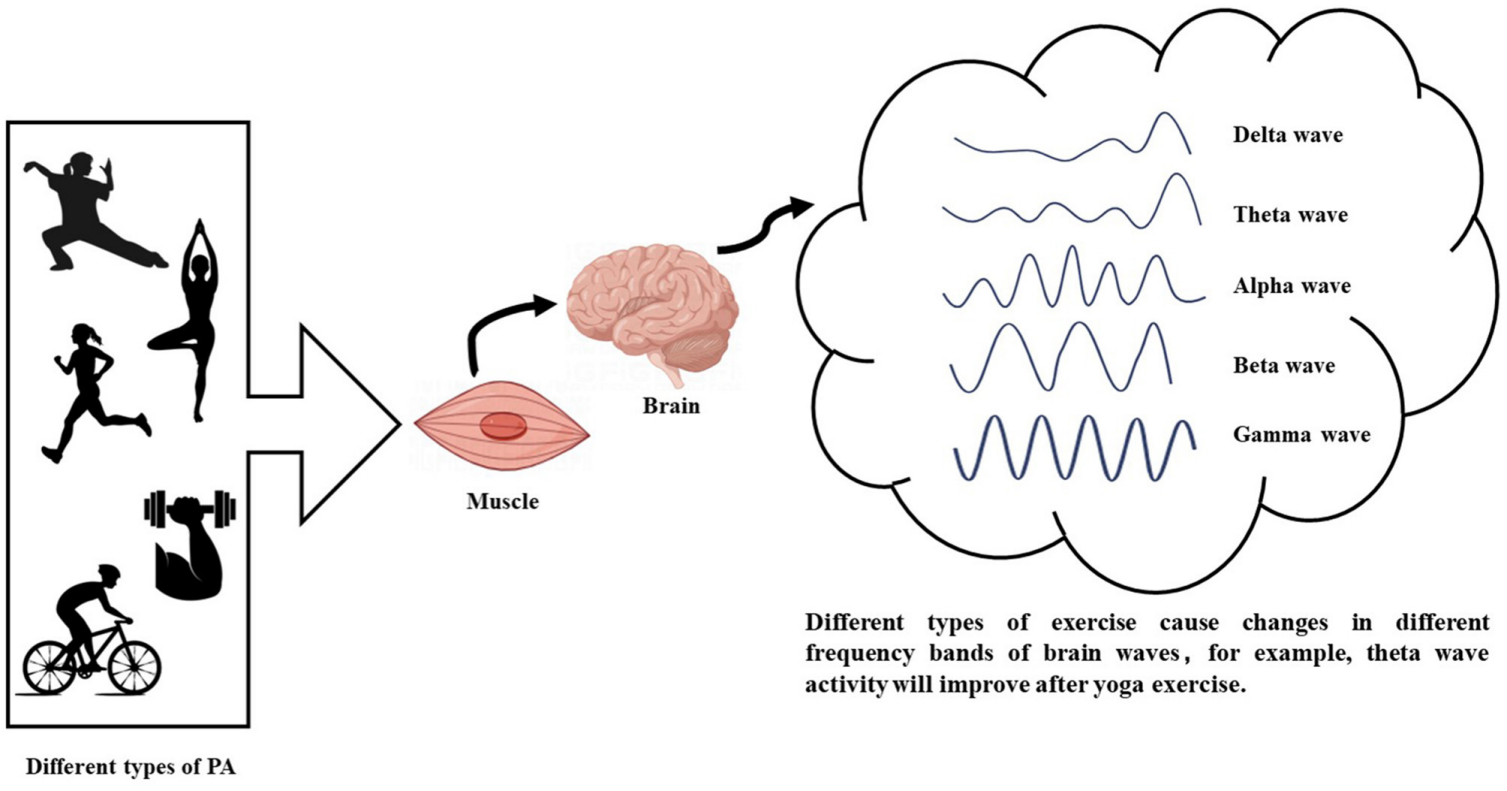
Different types of exercise affect different bands of brain waves.

Neuroplasticity is closely linked to neural oscillations, which interact and play a key role in brain function and adaptability (Tavano et al., 2023; Weiss et al., 2023). Oscillations are pivotal in regulating physiological processes during exercise, conscious perception, and cognitive functions (Van Ede et al., 2018). These oscillations are categorized into frequency bands, including delta (0.5–4 Hz), theta (4–8 Hz), alpha (8–13 Hz), beta (13–30 Hz), and gamma (30–100 Hz) waves (Steriade et al., 1990). By examining these neuronal rhythms, correlations between alterations in cognitive function and brain health can be established, offering insights into the effects of these oscillations on overall brain functioning (Figure 2). Despite conclusive evidence supporting the neuroprotective effects of exercise, whether each specific type of exercise entails distinct neuroprotective mechanisms remains debated in the realm of sports medicine (Ciria et al., 2018). Moreover, the comparative effectiveness of various exercise modalities remains contentious and requires further investigation (Liu et al., 2019).

Resistance training, defined as any physical activity involving the generation of muscular force against external resistance to enhance muscle size, strength, and endurance, is a fundamental component of physical fitness (Chow et al., 2021). Studies have established a correlation between low skeletal muscle mass or impaired muscle function and cognitive dysfunction (Oudbier et al., 2022; Peng et al., 2023). Neurological adaptations to resistance training, particularly observed within the first 6–8 weeks of initiating a regimen, often result in rapid strength gains even in the absence of muscle hypertrophy (Folland and Williams, 2007). Resistance exercise (RE) can activate various neurochemicals, including lactate, cortisol (COR), BDNF, norepinephrine, and dopamine (Hsieh et al., 2016). Harveson et al. (2016) reported the beneficial impact of acute moderate resistance exercise (70% 1RM) on working memory in male adults aged 21–30 and older adults aged 65-72. However, these health benefits of acute resistance exercise may be short-lived. Following acute isometric RE in the lower extremities, Basso and Suzuki (2017) observed a temporary elevation in peripheral blood levels of IGF-1, with cognitive benefits lasting up to 2 h post-exercise. Conversely, older adults engaged in 52 weeks of resistance exercise displayed increased peripheral blood IGF-1 levels and enhanced cognitive performance (Tsai et al., 2019). Moreover, skeletal muscles are known to secrete neurotrophic and muscle factors such as insulin-like growth factor-1 and BDNF, fostering structural and functional plasticity in brain regions like the hippocampus and prefrontal cortex (Broadhouse et al., 2020; Coutinho et al., 2022). This release of growth factors, not exclusive to resistance exercise, may be a key mechanism (Nicola et al., 2024). Long-term neural structure and function are likely supported by resistance exercise through this mechanism, potentially enhancing cognitive function through improved skills and muscle-related adaptations.

In recent years, researchers have endeavored to elucidate the relationship between resistance training and neural oscillations, aiming to provide a more comprehensive understanding of the effects of resistance exercise on brain function from alternative perspectives (Weiss et al., 2023). A study showed that a physical activity program for 4 months led to an increase in frontal alpha activity during an attention task, suggesting an improvement in the neural dynamics linked to visual attention (Chaire et al., 2020). Furthermore, studies have been conducted to determine how different exercise regimens affect muscle strength and cognitive performance. These studies demonstrate that brief, intense resistance training can positively affect neural oscillations and brain plasticity, resulting in improved cognitive performance and synaptic plasticity (Harrison et al., 2019; Nuzzo et al., 2024). In a separate investigation into the impact of resistance exercise therapy on individuals with mild cognitive impairment, a notable decline in theta band power was documented following 12 weeks of group resistance training (Chmielewski et al., 2021). While resistance training is increasingly used in clinical settings, more research is needed to determine the most effective dosage for optimal health effects.

In addition to resistance training, aerobic exercise represents another common form of PE. Comparative analyses of systematic reviews and meta-analyses from the past 5 years suggest that aerobic exercises such as walking, jogging, and cycling are more frequently employed in research aimed at improving cognitive health compared to resistance training (Lesinski et al., 2015; Sáez de Asteasu et al., 2017; Northey et al., 2018; Gavelin et al., 2021; Kwok et al., 2022). This preference may stem, in part, from the accessibility and simplicity of aerobic exercises. Aerobic activities are straightforward to perform, often requiring minimal specialized equipment and supervision, making them suitable for incorporation into daily routines, particularly for untrained individuals and those with cognitive impairments (Chow et al., 2021). Results from numerous studies involving humans and animals suggest that voluntary running in rodents or aerobic training in humans can enhance brain plasticity, including synaptogenesis, neurogenesis, and cognition (Falkai et al., 2017). Several meta-analyses focusing on adults aged over 55 have highlighted the beneficial effects of aerobic exercise on attention, processing speed, executive function, memory, and working memory (Falkai et al., 2017; Northey et al., 2018). Additionally, a study by Stern et al. found that 6 months of aerobic exercise positively impacted brain health in healthy individuals aged 20 to 67 years. A study on neural oscillations revealed that participants in the exercise group experienced an increase in frontal alpha power magnitude after a 4-month aerobic exercise program, as measured using the Visual Attention Search Task (VAS) (Chaire et al., 2020). In a study investigating the neural mechanisms of aerobic exercise-induced hyperalgesia, participants exhibited reduced pain scores in the tibialis anterior and rectus femoris muscles following moderate-intensity aerobic exercise, accompanied by increased power of alpha oscillations, indicative of central descending inhibition (Zheng et al., 2021). Decreases in task-related power within the beta band of sensorimotor regions, as measured by EEG, are believed to indicate heightened cortical activation and active processing of motor tasks (Gerloff et al., 1998; Engel and Fries, 2010). The alpha and beta bands experienced an increase in EEG coherence after a single session of aerobic exercise, with the highest level of improvement observed in the high beta band (Mark et al., 2023). The following are potential underlying mechanisms that can improve cognitive function through aerobic exercise.

Aerobic exercise has become the cornerstone of clinical management for various neurodegenerative diseases. However, the intensity of aerobic exercise has been a subject of controversy in research (Tsai et al., 2019; Sudo et al., 2022). In a study analyzing the effects of a single 25-min moderate-intensity exercise intervention in older adults, the experimental group exhibited improved motor performance on the force modulation task immediately post-exercise compared to the control group (Hübner et al., 2018). However, many studies indicate that high-intensity aerobic exercise [≥80% of maximal power output, ≥80% of maximal oxygen uptake (VO2)] may lead to brain and cognitive damage (Komiyama et al., 2020; Stone et al., 2020). Recently, a narrative review emphasized the impact of aerobic exercise on cerebral blood flow (CBF), cerebral oxygenation, and cerebral metabolism (Sudo et al., 2022). It was noted that there is a gradual increase in CBF during light to moderate-intensity exercise, whereas high-intensity exercise leads to a decrease in CBF. This suggests that the metabolic demands of the brain may not be met during high-intensity exercise, potentially negatively impacting cognition and brain health.

Even though overwhelming evidence suggests that physical exercise improves brain health and cognitive function, concerns remain. Sports-related concussions (SRCs) are frequently experienced by athletes due to biomechanical forces to the head, resulting in transient clinical signs, symptoms, and dysfunction (Chmielewski et al., 2021). EEG is often utilized clinically for diagnosing moderate to severe traumatic brain injury (TBI), monitoring acute and subacute neurophysiological changes, and serving as a prognostic indicator for the patient's clinical presentation and recovery (Corbin-Berrigan et al., 2023). It is increasingly recognized that excessive exercise could result in brain damage. While EEG is not the primary method for monitoring and treating sudden cardiac death (SCD) in clinical settings, it proves valuable for detecting potential abnormalities (Rapp et al., 2015; Kamins et al., 2017). EEG abnormalities have been identified in patients with SRC in some studies. Research conducted by Teel et al. (2014) revealed that concussed participants exhibited reduced alpha, beta, and theta bandwidths, particularly showing a significant reduction in beta power during computerized neurocognitive testing (ImPACT). In a study that gathered EEGs from concussed patients, analysis revealed alterations in the alpha and beta frequency bands that significantly correlated with Glasgow Coma Scale (GCS) scores (Frohlich et al., 2022). Specifically, higher GCS scores were associated with reduced alpha power and increased beta power. These findings highlight the importance of monitoring and analyzing oscillations in different frequency bands following injury for accurate diagnosis and prognosis in post-injury care (Frohlich et al., 2022). Caution should be exercised regarding excessive physical activity due to its potential adverse impact on brain health.

In recent years, researchers have shown increased interest in various forms of exercise due to the differing needs of diverse populations. Mind-body exercises such as yoga, Tai Chi, and dance have been utilized in the treatment of some chronic diseases. Further research on the effects of these types of exercises on brain health is necessary.

## 3 How mind-body exercise affects human neural activity and cognitive functioning

Recent studies have focused on the potential benefits of mind-body exercises, such as yoga, dance, and Tai Chi, on brain health (Gothe et al., 2019). Mind-body exercise, a form of physical exercise that utilizes a mind-body approach to achieve physical and mental benefits, has garnered significant attention from researchers (Kwok et al., 2019). Despite its lower intensity compared to other forms of exercise, mind-body exercise still offers specific health benefits that have piqued researchers' interest.

Dance is a multifaceted activity that includes physical exercise and cognitive, social, and artistic components, which are linked to visual-spatial, cognitive, and executive functions in individuals (Chirles et al., 2017; Karkou et al., 2019). It has been demonstrated that dance can enhance cognitive and executive functioning, especially in individuals with mild cognitive impairment (Qi et al., 2019; Zhu et al., 2020). A recent systematic review further supports the idea that dancing can lead to increased cognitive stimulation, promoting neuroplasticity in the brain (Teixeira-Machado et al., 2019). Dance interventions have been shown to positively impact brain plasticity, causing various structural changes, such as increases in gray matter volume and white matter integrity (Teixeira-Machado et al., 2019). An experiment was conducted by Qi et al. (2019) utilizing functional magnetic resonance imaging (fMRI) assessed changes in brain activity using the amplitude of low-frequency fluctuations (ALFF) metric. The results revealed that older adults with mild cognitive impairment who participated in the dance intervention group exhibited increased ALFF in brain regions, including the bilateral frontotemporal, insular, anterior cingulate, and parahippocampal cortex. Additionally, substantial cortical thickening was observed in specific regions of the right hemisphere in the dance intervention group (Rektorova et al., 2020). The positive impacts of dance interventions on brain structure and function suggest that improvements in cognitive functioning may be achievable through dance therapy (Wu et al., 2021). Notably, a study reported a significant increase in white matter volume in frontal and parietal regions, as well as in the corpus callosum, following a 6-month dance intervention (Voss et al., 2017a; Rektorova et al., 2020). The engagement of cognitive, somatosensory, and motor regions in complex dance movements can have subtle yet beneficial effects on cognitive functions (Wu et al., 2021). Dance therapy has also been credited with enhancing coordination between neurosensory and muscular systems, increasing flexibility, and muscular strength in both the upper and lower extremities (Douka et al., 2019; Chan et al., 2021). While existing studies have confirmed the positive health effects of dance interventions on both the brain and body, further research with larger sample sizes and extended interventions is warranted. This is due to the necessity of comprehensive instruction and coaching in dance, as well as the ongoing need to explore the underlying physiological mechanisms more extensively.

Yoga, considered a common mind-body exercise intervention alongside dancing, is a positive movement practice that allows individuals to move gradually and safely into physical postures while emphasizing relaxation, full breathing, and awareness of physical sensations and thoughts (Gothe et al., 2019). One study revealed that experienced yoga practitioners exhibited greater overall brain gray matter volume (GMV) compared to non-practitioners (Villemure et al., 2015). In a study conducted over 3 months by Krause-Sorio et al. (2022) older women at risk for Alzheimer's disease who engaged in weekly yoga training and completed assignments punctually demonstrated retention of GMV across all brain regions, as well as increased volumes in the left precentral cortex and lateral occipital cortex. Several studies have examined the effectiveness of combining transcranial direct current stimulation (tDCS) with yoga interventions. These studies have shown that the combination of yoga and active tDCS leads to improved subnetwork connectivity across all EEG frequency bands (Sefat et al., 2022). These results hint at the possible physiological mechanisms underlying yoga's cognitive benefits. Recent recognition of yoga as a safe practice with positive cognitive effects in healthy older adults, those with mild cognitive impairment (MCI), and early-stage dementia patients is noteworthy (Brenes et al., 2019; Chobe et al., 2020). A randomized controlled trial revealed that yoga had a moderately significant impact on cognition, with attention and processing speed, executive function, and memory ranking as the top three affected domains (Voss et al., 2023). Although comparing the effects of various yoga interventions remains challenging due to the diversity in Hatha Yoga styles, these discrepancies could open new avenues for future research as they may lead to varying impacts on the nervous system (Voss et al., 2023). The growing recognition of yoga as an effective exercise intervention, along with ongoing exploration of its therapeutic effects on neurological disorders, points to the potential for optimized outcomes when combined with clinical treatments.

In recent years, the growing popularity of Tai Chi Chuan (TCC) as a physical and mental exercise can be attributed to its multifaceted health benefits (Wayne et al., 2014). Originating in China during the 17^th^ century AD (Yang et al., 2022). TCC has been linked to positive changes in brain function and structure in various studies. For instance, a study involving a 40-week TCC intervention revealed a significant increase in brain volume (Yang et al., 2022). Furthermore, a functional magnetic resonance imaging (fMRI) study showed that a 12-week TCC intervention led to improvements in subjects' low-frequency fluctuation (fALFF) scores in the lateral prefrontal cortex compared to a control intervention (Yue et al., 2020). Such enhancements in specific brain regions can potentially alleviate age-related memory loss. Notably, older women with 6 years of Tai Chi experience demonstrated greater homogeneous activation of spontaneous regions in temporal areas, including the fusiform gyrus and hippocampus, compared to those engaging in 6 years of walking as a control intervention, as indicated by another fMRI study (Yue et al., 2020). Moreover, systematic evaluations and meta-analyses have underscored the positive impact of TCC on overall cognitive functioning, memory, learning, and visual perception among patients with cognitive impairment (Yang et al., 2020). However, experimental studies have failed to establish the superiority of TCC over control interventions in improving depressive symptoms or executive function (Wei et al., 2022). There is a lack of conclusive evidence supporting the notion that Tai Chi is more effective in addressing these features than alternative control methods. This underscores the need for further research to elucidate the precise benefits of TCC on physical and mental health, along with the underlying neural mechanisms supporting these effects.

With increasing interest in mind-body exercises such as yoga and Tai Chi, which offer the flexibility to adjust exercise loads and cater to a wider range of individuals, including the elderly and those with neurological conditions, it is evident that these exercises are becoming more popular. The debate surrounding adaptive movement patterns persists due to the insufficient availability of reliable monitoring instruments (Chaire et al., 2020). Therefore, it is crucial to conduct further research to elucidate the underlying neural mechanisms, necessitating a better understanding of their impact (Yang et al., 2020; Wei et al., 2022).

## 4 Age-related effects of PE on brain health and neurodegenerative disease rehabilitation via neural oscillation mechanisms

The advent of neuroimaging technology has enabled the non-invasive monitoring of human brain activity. However, these methods have inherent limitations that hinder the exploration of intricate neural mechanisms. Factors such as low amplitude, artifacts, and resistance to wearing electrode caps can adversely affect the accuracy of EEG signals (Baumgartner and Koren, 2018). Additionally, age (Borhani et al., 2021), health conditions (Weiss et al., 2023), and other factors can impact brain health and function. The role of neural oscillations and PE in brain health requires further research.

Changes in structural brain functioning linked to age-related cognitive decline, also known as normal cognitive aging, have been well-documented (Jafari et al., 2020). Individuals undergoing physiological aging often demonstrate increased power of low-frequency oscillations and a decrease and slowing of alpha activity, as observed in resting-state EEG (rsEEG) studies (Nobukawa et al., 2019). Consistent findings have shown shifts in oscillatory activity from posterior to anterior regions in older adults, characterized by an increase in frontal activity and a decrease in occipital activity (Perinelli et al., 2022). Specifically, the power spectral density (PSD) tends to plateau at 2–24 Hz in older adults compared to their younger counterparts, indicating alterations in brain activity with age (Voytek et al., 2015). Research has revealed that both slow and fast gamma power decrease with age, with a more pronounced decrease observed in fast gamma power (Murty et al., 2020). This decline in gamma power was further corroborated by another study, which showed age-related reductions in gamma power in the motor region, suggesting the generalizability of this phenomenon across various brain regions (Gaetz et al., 2020). Given the known involvement of the gamma band in higher cognitive functions such as attention and working memory, the observed changes in gamma band activity in older adults align with the hypothesis that cognitive decline is associated with aging (Murty et al., 2020). Moreover, a substantial relationship between memory retrieval accuracy and rsEEG band power has been established. Increases in alpha and beta bands in the right parietal and right frontal lobes have been found to be significantly associated with decreased memory retrieval accuracy (Borhani et al., 2021). These findings underscore the importance of understanding the neural mechanisms underlying age-related cognitive changes and highlight the potential utility of rsEEG measures in predicting cognitive performance in older adults.

**Figure 3:**
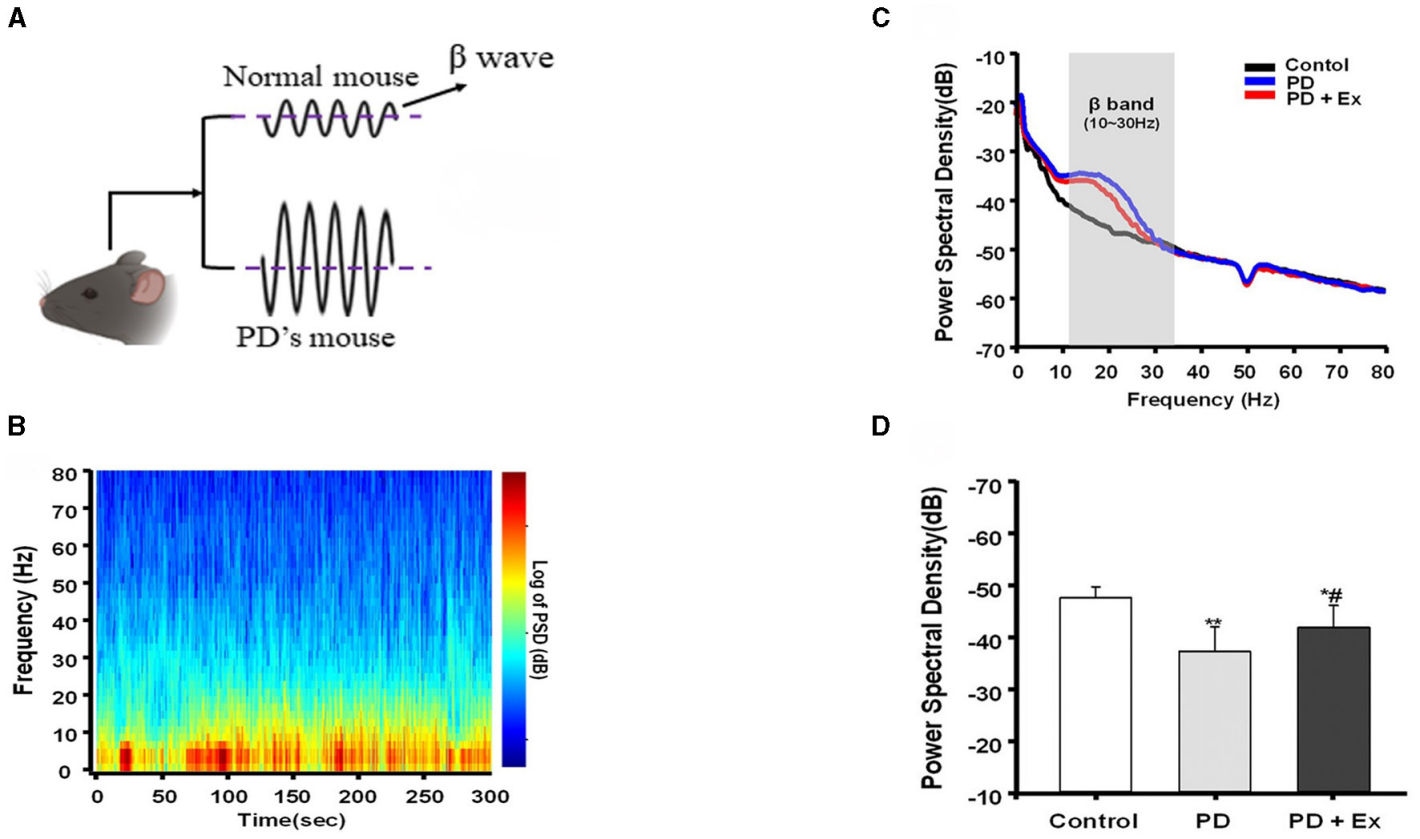
Beta burst dynamics in PD. Increased β pulse amplitude appears in PD mouse model **(A)**. β bursts mitigated by exercise intervention (Yu et al., 2021). **(B–D)** Are replotted based on Liu (2021). A representative fast Fourier transform (FFT)-based spectrogram depicts the time-frequency spectral power of motor cortex LFPs during 5-min epochs of rest **(B)**. Linear graphs show averaged LFP power (0–80 Hz) spectra for the Control, Parkinson's disease (PD), and PD + exercise (Ex) groups **(C)**, with the gray area showing that the beta band in the 10–30 Hz range of LFP power was significantly increased in 6-hydroxydopamine (6-OHDA)-lesioned rats. The average PSDs of the three groups are shown in **(D)**. ^*^P < 0.05, ^**^P < 0.01; and compared with the PD group, ^#^*P* < 0.05.

The risk of developing neurodegenerative diseases like Parkinson's disease (PD) and Alzheimer's disease (AD) increases with age (Mattson and Arumugam, 2018). PD is a prevalent neurodegenerative disorder clinically characterized as a “movement disorder” due to symptoms such as stiffness and bradykinesia. Excessive activity in the beta band is a hallmark of basal ganglia signaling in PD patients, characterized by sudden bursts rather than continuous elevation. Prior studies have elucidated that prolonged beta bursts in PD contribute to heightened oscillatory synchronization within the subthalamic nucleus (STN), impeding the encoding capacity of local circuits (Tinkhauser et al., 2018). Consequently, researchers have probed whether animal models of PD exhibit similar alterations in beta activity within the basal ganglia following dopamine loss and resultant motor dysfunction. This exploration has facilitated direct comparisons of synchronized activity across various behaviors between control and dopamine-deprived groups (Avila et al., 2010), illuminating the origins and ramifications of synchronous increases in basal ganglia output within the beta range (Figure 3). One study discovered that bilateral electrodes implanted in the substantia nigra pars reticulata (SNpr) of rats with unilateral damage revealed a correlation between dopamine loss and heightened spike activity in both local field potential (LFP) power and beta frequency ranges (Johansson et al., 2022).

The surge in activity was predominantly observed in the high-frequency range while the rats walked on a circular treadmill, with the most pronounced activity occurring in the low-frequency range during periods of inattentive rest (Avila et al., 2010). Although the experimental design of the rodent study could not precisely determine the timing of muscle activity following reawakening from a state of inattentive rest, it facilitated a direct comparison between the damaged and undamaged hemispheres during complex and sustained exercise (Avila et al., 2010). The study indicated that the loss of dopamine significantly impacted the synchronization of output from the basal ganglia beta region in rats walking on a treadmill. Studies conducted on semi-Parkinsonian rats have demonstrated that dopamine depletion is linked to increased expression of low-frequency activities during rest, which diminishes with exercise (Palasz et al., 2019). These results suggest that abnormal beta wave activity is associated with impaired motor function and that exercise interventions in laboratory animals can modulate beta wave activity (Tinkhauser et al., 2018; Palasz et al., 2019). This presents a novel avenue for the treatment and monitoring of patients with Parkinson's disease.

AD is another common degenerative disease that, like Parkinson's disease (PD), becomes more prevalent with age. Gamma oscillations, which are rhythmic fluctuations of local field potentials (LFPs) with a frequency range of approximately 25–100 Hz, are a notable feature in various brain regions, such as the hippocampus (Mably and Colgin, 2018). These oscillations are believed to be involved in attention selection and memory processes (Bieri et al., 2014). Studies have shown that the amplitude of slow gamma oscillations tends to increase during the proper execution of tasks involving associative memory, particularly when cue-induced memory retrieval is anticipated. As a result, researchers hypothesize that certain cognitive disorders associated with brain diseases may be linked to disturbances in gamma rhythms (Bieri et al., 2014; Mehak et al., 2022).

Findings from a study revealed that 3xTg mice exhibited a decrease in the slow gamma power of the hippocampus CA1 within a familiar circular orbit, alongside an unstable spatial representation of CA1 place cells, and a reduction in the slow gamma coordination of CA1 place cells' discharge (Mably et al., 2017). This slow gamma impairment could potentially lead to the incomplete retrieval of stored spatial information from CA3 to CA1. These results suggest that targeting slow gamma interference could offer a promising new avenue for addressing memory deficits in Alzheimer's disease (Booth et al., 2016). It has been demonstrated that exercise enhances neurotrophic factors, growth factors, and synaptic markers, and reduces neuroinflammation, making it a key strategy for both the prevention and treatment of Alzheimer's disease, often in conjunction with medications (Cotman et al., 2007; Cho et al., 2015). In a study combining 40 Hz light stimulation with exercise in the 3xTg mouse model, significant improvements were observed after a 12-week intervention period (Park et al., 2020). These included a reduction in tau phosphorylation and Aβ levels in the hippocampus, as well as enhancements in spatial learning, memory, long-term memory, mitochondrial function, and neuroplasticity. Although this study did not directly measure gamma oscillations, previous research has shown that alterations in hippocampal gamma oscillations occur over time and with Aβ levels (Kurudenkandy et al., 2014), with the 3xTg-AD model displaying abnormal synchronization of beta and gamma frequencies (Castano-Prat et al., 2019).

The lack of clinically recognized medications or medical treatments for degenerative diseases has propelled exercise to the forefront as a key intervention method (De la Rosa et al., 2020). Long-term exercise training has beneficial effects on delaying physiological memory loss, making it an effective strategy for preventing age-related memory loss and neurodegeneration (De la Rosa et al., 2020). One study demonstrated that 6 months of exercise (60 min per session, 3 days per week) increased gray and white matter in the anterior cingulate cortex in cognitively healthy older adults, as measured by magnetic resonance imaging (Sebastián-Romagosa et al., 2020). Through the analysis of EEG signals, exercise-induced changes can be objectively assessed, enabling the examination of physiological transformations in the brain (Albert et al., 2011). Mild cognitive impairment (MCI) represents a transitional phase between normal aging and early dementia, characterized by a decline in certain cognitive functions (Albert et al., 2011). Clinical research has shown that prolonged exercise training over a period of 6 to 12 weeks can lead to a reduction in delta and theta band power, along with an elevation in beta and alpha band power, as well as increased EEG complexity and connectivity in patients with MCI (Pedroso et al., 2021). These findings suggest that cortical activity and cognition in individuals with MCI may be improved by exercise, as evidenced by these observed alterations.

Animal model experiments also showed the benefits of PE. In a transgenic mouse model of AD, voluntary and forced exercise interventions led to a decrease in amyloid-beta (Aβ) plaques and neurofibrillary tangles (NFTs). Improvements in learning and memory were correlated with these findings in some cases (Ohia-Nwoko et al., 2014; Tapia-Rojas et al., 2015). A recent study found that treadmill exercise for 12 weeks as part of an AD intervention program increased mitochondrial proteostasis in mice. Concurrently, the exercised mice exhibited a marked decrease in escape latency and a significant increase in crossings over the platforms in the water maze test (Cui et al., 2023). Research has shown that 4 weeks of sustained platform-running exercise can positively impact the plasticity of motor cortex function in rats with PD (Liu, 2021). Specifically, this exercise regimen led to the amelioration of abnormal neural activity in the primary motor cortex (M1) region of PD rats. The disruption of M1 neurons and beta oscillations due to the exercise intervention was evidenced by changes in oscillatory patterns (Liu, 2021). A notable finding was the decrease in spiking phase locking of beta band oscillations observed in the motor intervention group compared to the PD group. Additionally, voluntary running exercise has been found to enhance BDNF expression in the dorsal striatum of PD model mice (Bastioli et al., 2022). This study also found a notable increase in extracellular dopamine concentrations in the striatum of the exercise group. The importance of dopamine (DA) in exercise, motor learning, reward, motivation, and emotion cannot be overstated (Schultz et al., 2017; Athalye et al., 2018). Exercise has the potential to alleviate motor deficits in PD by slowing down the neurodegeneration of DA neurons (Bastioli et al., 2022). These studies suggest that exercise plays a pivotal role in the prevention and treatment of neurodegenerative diseases, demonstrating positive effects on behavior and brain plasticity in both animal and clinical trials (Cotman et al., 2007; Avila et al., 2010; Kurudenkandy et al., 2014; Tinkhauser et al., 2018; Park et al., 2020; Lee et al., 2021).

As individuals age, their brains undergo several changes that can lead to cognitive decline and slowed responses (Borhani et al., 2021). These changes, although subtle, can have a lasting and significant impact on an older person's life (Machado et al., 2019). Despite the absence of pharmaceutical agents specifically recommended for treating and preventing this degenerative process, exercise emerges as a potentially effective and accessible alternative. Monitoring changes in the brain's oscillations allows for timely adjustments to the exercise program (Wan et al., 2024). While numerous studies have highlighted the benefits of exercise on brain health and cognitive functioning, some have reported a weak association between exercise and cognitive performance (Iso-Markku et al., 2024). To achieve superior intervention outcomes, it is imperative to identify more effective methods to supplement single exercise interventions.

## 5 Conclusion and future direction

As previously highlighted, abundant evidence supports the significant impact of exercise on brain health, including enhancements in cognitive abilities, alterations in neuronal oscillatory activity, and other mechanisms across the lifespan in both healthy and pathological conditions. However, variations in task paradigms, age, gender, intervention duration, exercise type, and other protocols introduce considerable heterogeneity among studies, sometimes leading to conflicting results regarding the exercise-brain relationship (Browne et al., 2017; Vanderbeken and Kerckhofs, 2017; Gallardo-Gómez et al., 2022). For instance, a recent systematic review and meta-analysis in humans concluded that there may not be a linear association between exercise and cognition, particularly among older adults (Gallardo-Gómez et al., 2022). This study also revealed differential dose-response relationships for various exercise modalities, with resistance training potentially offering superior efficacy compared to other forms (Gallardo-Gómez et al., 2022). Against this backdrop, the standardization of research methodologies in this field is paramount (Augusto-Oliveira et al., 2023). This entails the harmonization of exercise protocols, including modality, design, intensity, and duration, as well as consideration of the timing of interventions relative to injury or neurodegenerative disease diagnosis and the characteristics of the study population. Essential aspects to be addressed include interference controls, cognitive function assessment methodologies, monitoring techniques, and the timing of outcome evaluations. Moreover, increasing sample sizes, particularly in human studies, is imperative due to the substantial heterogeneity among individuals, necessitating larger cohorts for robust conclusions and result replication (Augusto-Oliveira et al., 2023).

Combinations of different exercise modalities have garnered attention in research due to their reported effects on brain health and neural oscillations (Gothe et al., 2019; Yang et al., 2020). Identifying specific subgroups of individuals who stand to benefit most from particular physical activities is essential for tailoring personalized exercise regimens based on individual or group characteristics. Evolving data acquisition technology now allows for the simultaneous acquisition of EEG and EMG signals, enhancing the feasibility of research. This simultaneous capture during exercise enhances researchers' understanding of the muscle-brain relationship and facilitates the exploration of the deeper connections between physical activity and neural processes. Known as corticomuscular coherence (CMC), the coupling of sensorimotor cortical rhythms and muscle activity serves as a fundamental aspect of this investigation (Bourguignon et al., 2019). Through the analysis of EEG and EMG signals during movement, researchers can delve into the intricate relationship between the brain and physical motion.

In summary, physical activity is a crucial approach to preserving health and combating cognitive decline and neurodegenerative disorders. The importance of these variables when developing a personalized exercise prescription is due to the potential for disparate health effects based on the specific type and intensity of exercise. During exercise, neural oscillation changes provide new insights into monitoring the therapeutic effectiveness of PE and making prompt adjustments to the exercise program. Future studies may employ simultaneous brain and electromyography acquisition to further probe the nuanced interplay between exercise and brain functions. This knowledge holds considerable potential for advancing the development of precise non-pharmacological interventions aimed at enhancing brain health, preventing related diseases, and informing the creation of evidence-based personalized exercise prescriptions.

## Author contributions

XL: Writing – original draft. XQ: Writing – original draft. KS: Writing – review & editing. YY: Writing – review & editing. JS: Writing – review & editing.

## References

[B1] Albert M. S. DeKosky S. T. Dickson D. Dubois B. Feldman H. H. Fox N. C.. (2011). The diagnosis of mild cognitive impairment due to Alzheimer's disease: recommendations from the National Institute on Aging-Alzheimer's Association workgroups on diagnostic guidelines for Alzheimer's disease. Alzheimers. Dement. 7, 270–279. 10.1016/j.jalz.2011.03.008 21514249 PMC3312027

[B2] Athalye V. R. Santos F. J. Carmena J. M. Costa R. M. (2018). Evidence for a neural law of effect. Science 359, 1024–1029. 10.1126/science.aao6058 29496877

[B3] Augusto-Oliveira M. Arrifano G. P. Leal-Nazaré C. G. Santos-Sacramento L. Lopes-Araújo A. Royes L. F. F.. (2023). Exercise reshapes the brain: molecular, cellular, and structural changes associated with cognitive improvements. Mol. Neurobiol. 60, 6950–6974. 10.1007/s12035-023-03492-8 37518829

[B4] Avila I. Parr-Brownlie L. C. Brazhnik E. Castañeda E. Bergstrom D. A. Walters J. R. (2010). Beta frequency synchronization in basal ganglia output during rest and walk in a hemiparkinsonian rat. Exp. Neurol. 221, 307–319. 10.1016/j.expneurol.2009.11.016 19948166 PMC3384738

[B5] Basso J. C. Suzuki W. A. (2017). The effects of acute exercise on mood, cognition, neurophysiology, and neurochemical pathways: a review. Brain Plast 2, 127–152. 10.3233/BPL-160040 29765853 PMC5928534

[B6] Bastioli G. Arnold J. C. Mancini M. Mar A. C. Gamallo-Lana B. Saadipour K.. (2022). Voluntary exercise boosts striatal dopamine release: evidence for the necessary and sufficient role of BDNF. J. Neurosci. 42, 4725–4736. 10.1523/JNEUROSCI.2273-21.2022 35577554 PMC9186798

[B7] Baumgartner C. Koren J. P. (2018). Seizure detection using scalp-EEG. Epilepsia 59, 14–22. 10.1111/epi.14052 29873826

[B8] Bieri K. W. Bobbitt K. N. Colgin L. L. (2014). Slow and fast γ rhythms coordinate different spatial coding modes in hippocampal place cells. Neuron 82, 670–681. 10.1016/j.neuron.2014.03.013 24746420 PMC4109650

[B9] Booth C. A. Witton J. Nowacki J. Tsaneva-Atanasova K. Jones M. W. Randall A. D.. (2016). Altered intrinsic pyramidal neuron properties and pathway-specific synaptic dysfunction underlie aberrant hippocampal network function in a mouse model of tauopathy. J. Neurosci. 36, 350–363. 10.1523/JNEUROSCI.2151-15.2016 26758828 PMC4710765

[B10] Borhani S. Zhao X. Kelly M. R. Gottschalk K. E. Yuan F. Jicha G. A.. (2021). Gauging working memory capacity from differential resting brain oscillations in older individuals with a wearable device. Front. Aging Neurosci. 13:625006. 10.3389/fnagi.2021.625006 33716711 PMC7944100

[B11] Bourguignon M. Jousmäki V. Dalal S. S. Jerbi K. De Tiège X. (2019). Coupling between human brain activity and body movements: Insights from non-invasive electromagnetic recordings. Neuroimage 203:116177. 10.1016/j.neuroimage.2019.116177 31513941

[B12] Brenes G. A. Sohl S. Wells R. E. Befus D. Campos C. L. Danhauer S. C. (2019). The Effects of Yoga on Patients with Mild Cognitive Impairment and Dementia: A Scoping Review. Am. J. Geriatr. Psychiatry 27, 188–197. 10.1016/j.jagp.2018.10.013 30413292 PMC6541218

[B13] Broadhouse K. M. Singh M. F. Suo C. Gates N. Wen W. Brodaty H.. (2020). Hippocampal plasticity underpins long-term cognitive gains from resistance exercise in MCI. Neuroimage Clin. 25:102182. 10.1016/j.nicl.2020.102182 31978826 PMC6974789

[B14] Browne S. E. Flynn M. J. O'Neill B. V. Howatson G. Bell P. G. Haskell-Ramsay C. F. (2017). Effects of acute high-intensity exercise on cognitive performance in trained individuals: a systematic review. Prog. Brain Res. 234, 161–187. 10.1016/bs.pbr.2017.06.003 29031462

[B15] Caspersen C. J. Powell K. E. Christenson G. M. (1985). Physical activity, exercise, and physical fitness: definitions and distinctions for health-related research. Public Health Rep. 100, 126–131. 3920711 PMC1424733

[B16] Castano-Prat P. Perez-Mendez L. Perez-Zabalza M. Sanfeliu C. Giménez-Llort L. Sanchez-Vives M. V. (2019). Altered slow (< 1 Hz) and fast (beta and gamma) neocortical oscillations in the 3xTg-AD mouse model of Alzheimer's disease under anesthesia. Neurobiol. Aging 79, 142–151. 10.1016/j.neurobiolaging.2019.02.009 31103943

[B17] Chaire A. Becke A. Düzel E. (2020). Effects of physical exercise on working memory and attention-related neural oscillations. Front. Neurosci. 14:239. 10.3389/fnins.2020.00239 32296302 PMC7136837

[B18] Chan C. H. Y. Ji X.-W. Chan J. S. M. Lau B. H. P. So K.-F. Li A.. (2017). Effects of the integrative mind-body intervention on depression, sleep disturbances and plasma IL-6. Psychother. Psychosom. 86, 54–56. 10.1159/000447541 27884001

[B19] Chan J. K. Y. Klainin-Yobas P. Chi Y. Gan J. K. E. Chow G. Wu X. V. (2021). The effectiveness of e-interventions on fall, neuromuscular functions and quality of life in community-dwelling older adults: a systematic review and meta-analysis. Int. J. Nurs. Stud. 113:103784. 10.1016/j.ijnurstu.2020.103784 33120138

[B20] Chirles T. J. Reiter K. Weiss L. R. Alfini A. J. Nielson K. A. Smith J. C. (2017). Exercise training and functional connectivity changes in mild cognitive impairment and healthy elders. J. Alzheimers. Dis. 57, 845–856. 10.3233/JAD-161151 28304298 PMC6472271

[B21] Chmielewski T. L. Tatman J. Suzuki S. Horodyski M. Reisman D. S. Bauer R. M.. (2021). Impaired motor control after sport-related concussion could increase risk for musculoskeletal injury: implications for clinical management and rehabilitation. J. Sport Health Sci. 10, 154–161. 10.1016/j.jshs.2020.11.005 33188963 PMC7987572

[B22] Cho J. Shin M.-K. Kim D. Lee I. Kim S. Kang H. (2015). Treadmill running reverses cognitive declines due to Alzheimer disease. Med. Sci. Sports Exerc. 47, 1814–1824. 10.1249/MSS.0000000000000612 25574797

[B23] Chobe S. Chobe M. Metri K. Patra S. K. Nagaratna R. (2020). Impact of Yoga on cognition and mental health among elderly: a systematic review. Complement. Ther. Med. 52:102421. 10.1016/j.ctim.2020.102421 32951703

[B24] Chow Z.-S. Moreland A. T. Macpherson H. Teo W.-P. (2021). The central mechanisms of resistance training and its effects on cognitive function. Sports Med. 51, 2483–2506. 10.1007/s40279-021-01535-5 34417978

[B25] Ciria L. F. Perakakis P. Luque-Casado A. Sanabria D. (2018). Physical exercise increases overall brain oscillatory activity but does not influence inhibitory control in young adults. Neuroimage 181, 203–210. 10.1016/j.neuroimage.2018.07.009 29981904

[B26] Corbin-Berrigan L.-A. Teel E. Vinet S.-A. De Koninck B. P. Guay S.. (2023). The use of electroencephalography as an informative tool in assisting early clinical management after sport-related concussion: a systematic review. Neuropsychol. Rev. 33, 144–159. 10.1007/s11065-020-09442-8 32577950

[B27] Cotman C. W. Berchtold N. C. Christie L.-A. (2007). Exercise builds brain health: key roles of growth factor cascades and inflammation. Trends Neurosci. 30, 464–472. 10.1016/j.tins.2007.06.011 17765329

[B28] Coutinho L. A. Leão L. L. Cassilhas R. C. de Paula A. M. B. Deslandes A. C. Monteiro-Junior R. S. (2022). Alzheimer's disease genes and proteins associated with resistance and aerobic training: an in silico analysis. Exp. Gerontol. 168:111948. 10.1016/j.exger.2022.111948 36087875

[B29] Cui K. Li C. Fang G. (2023). Aerobic exercise delays alzheimer's disease by regulating mitochondrial proteostasis in the cerebral cortex and hippocampus. Life (Basel) 13:1204. 10.3390/life13051204 37240849 PMC10223061

[B30] De la Rosa A. Olaso-Gonzalez G. Arc-Chagnaud C. Millan F. Salvador-Pascual A. García-Lucerga C.. (2020). Physical exercise in the prevention and treatment of Alzheimer's disease. J. Sport Health Sci. 9, 394–404. 10.1016/j.jshs.2020.01.004 32780691 PMC7498620

[B31] Douka S. Zilidou V. I. Lilou O. Tsolaki M. (2019). Greek traditional dances: a way to support intellectual, psychological, and motor functions in senior citizens at risk of neurodegeneration. Front. Aging Neurosci. 11:6. 10.3389/fnagi.2019.00006 30740051 PMC6356054

[B32] El-Sayes J. Harasym D. Turco C. V. Locke M. B. Nelson A. J. (2019). Exercise-induced neuroplasticity: a mechanistic model and prospects for promoting plasticity. Neuroscientist 25, 65–85. 10.1177/1073858418771538 29683026

[B33] Engel A. K. Fries P. (2010). Beta-band oscillations–signalling the status quo? Curr. Opin. Neurobiol. 20, 156–165. 10.1016/j.conb.2010.02.015 20359884

[B34] Falkai P. Malchow B. Schmitt A. (2017). Aerobic exercise and its effects on cognition in schizophrenia. Curr. Opin. Psychiatry 30, 171–175. 10.1097/YCO.0000000000000326 28230631

[B35] Folland J. P. Williams A. G. (2007). The adaptations to strength training : morphological and neurological contributions to increased strength. Sports Med. 37, 145–168. 10.2165/00007256-200737020-00004 17241104

[B36] Frohlich J. Crone J. S. Johnson M. A. Lutkenhoff E. S. Spivak N. M. Dell'Italia J.. (2022). Neural oscillations track recovery of consciousness in acute traumatic brain injury patients. Hum. Brain Mapp. 43, 1804–1820. 10.1002/hbm.25725 35076993 PMC8933330

[B37] Gaetz W. Rhodes E. Bloy L. Blaskey L. Jackel C. R. Brodkin E. S.. (2020). Evaluating motor cortical oscillations and age-related change in autism spectrum disorder. Neuroimage 207:116349. 10.1016/j.neuroimage.2019.116349 31726253

[B38] Gallardo-Gómez D. Del Pozo-Cruz J. Noetel M. Álvarez-Barbosa F. Alfonso-Rosa R. M. Del Pozo Cruz B. (2022). Optimal dose and type of exercise to improve cognitive function in older adults: a systematic review and bayesian model-based network meta-analysis of RCTs. Ageing Res. Rev. 76:101591. 10.1016/j.arr.2022.101591 35182742

[B39] Gavelin H. M. Dong C. Minkov R. Bahar-Fuchs A. Ellis K. A. Lautenschlager N. T.. (2021). Combined physical and cognitive training for older adults with and without cognitive impairment: a systematic review and network meta-analysis of randomized controlled trials. Ageing Res. Rev. 66:101232. 10.1016/j.arr.2020.101232 33249177

[B40] Gerloff C. Richard J. Hadley J. Schulman A. E. Honda M. Hallett M. (1998). Functional coupling and regional activation of human cortical motor areas during simple, internally paced and externally paced finger movements. Brain 121, 1513–1531. 10.1093/brain/121.8.1513 9712013

[B41] Gothe N. P. Khan I. Hayes J. Erlenbach E. Damoiseaux J. S. (2019). Yoga effects on brain health: a systematic review of the current literature. Brain Plast 5, 105–122. 10.3233/BPL-190084 31970064 PMC6971819

[B42] Guo S. Huang Y. Zhang Y. Huang H. Hong S. Liu T. (2020). Impacts of exercise interventions on different diseases and organ functions in mice. J. Sport Health Sci. 9, 53–73. 10.1016/j.jshs.2019.07.004 31921481 PMC6943779

[B43] Han C. (2023). The oscillating mystery: The effects of forty-hertz entrainment in treating Alzheimer's disease. Brain-X 1:e14. 10.1002/brx2.14

[B44] Han C. Wang T. Wu Y. Li Y. Yang Y. Li L.. (2021a). The generation and modulation of distinct gamma oscillations with local, horizontal, and feedback connections in the primary visual cortex: a model study on large-scale networks. Neural Plast. 2021, 1–17. 10.1155/2021/8874516 33531893 PMC7834828

[B45] Han C. Wang T. Yang Y. Wu Y. Li Y. Dai W.. (2021b). Multiple gamma rhythms carry distinct spatial frequency information in primary visual cortex. PLoS Biol. 19:e3001466. 10.1371/journal.pbio.3001466 34932558 PMC8691622

[B46] Harrison P. W. James L. P. McGuigan M. R. Jenkins D. G. Kelly V. G. (2019). Resistance priming to enhance neuromuscular performance in sport: evidence, potential mechanisms and directions for future research. Sports Med. 49, 1499–1514. 10.1007/s40279-019-01136-3 31203499

[B47] Harveson A. T. Hannon J. C. Brusseau T. A. Podlog L. Papadopoulos C. Durrant L. H.. (2016). Acute effects of 30 minutes resistance and aerobic exercise on cognition in a high school sample. Res. Q. Exerc. Sport 87, 214–220. 10.1080/02701367.2016.1146943 26958898

[B48] Hosang L. Mouchlianitis E. Guérin S. M. R. Karageorghis C. I. (2022). Effects of exercise on electroencephalography-recorded neural oscillations: a systematic review. Int. Rev. Sport Exerc. Psychol. 2022, 1–54. 10.1080/1750984X.2022.2103841

[B49] Hsieh S.-S. Chang Y.-K. Hung T.-M. Fang C.-L. (2016). The effects of acute resistance exercise on young and older males' working memory. Psychol. Sport Exerc. 22, 286–293. 10.1016/j.psychsport.2015.09.004

[B50] Hübner L. Godde B. Voelcker-Rehage C. (2018). Acute exercise as an intervention to trigger motor performance and EEG beta activity in older adults. Neural Plast. 2018:4756785. 10.1155/2018/4756785 30675151 PMC6323490

[B51] Iso-Markku P. Aaltonen S. Kujala U. M. Halme H.-L. Phipps D. Knittle K.. (2024). Physical activity and cognitive decline among older adults: a systematic review and meta-analysis. JAMA Netw. Open 7:e2354285. 10.1001/jamanetworkopen.2023.54285 38300618 PMC10835510

[B52] Jafari Z. Kolb B. E. Mohajerani M. H. (2020). Neural oscillations and brain stimulation in Alzheimer's disease. Prog. Neurobiol. 194:101878. 10.1016/j.pneurobio.2020.101878 32615147

[B53] Johansson M. E. Cameron I. G. M. Van Der Kolk N. M. De Vries N. M. Klimars E. Toni I.. (2022). Aerobic exercise alters brain function and structure in parkinson's disease: a randomized controlled trial. Ann. Neurol. 91, 203–216. 10.1002/ana.26291 34951063 PMC9306840

[B54] Kamins J. Bigler E. Covassin T. Henry L. Kemp S. Leddy J. J.. (2017). What is the physiological time to recovery after concussion? A systematic review. Br. J. Sports Med. 51, 935–940. 10.1136/bjsports-2016-097464 28455363

[B55] Karkou V. Aithal S. Zubala A. Meekums B. (2019). Effectiveness of dance movement therapy in the treatment of adults with depression: a systematic review with meta-analyses. Front. Psychol. 10. 10.3389/fpsyg.2019.00936 31130889 PMC6509172

[B56] Komiyama T. Tanoue Y. Sudo M. Costello J. T. Uehara Y. Higaki Y.. (2020). Cognitive impairment during high-intensity exercise: influence of cerebral blood flow. Med. Sci. Sports Exerc. 52, 561–568. 10.1249/MSS.0000000000002183 31609297

[B57] Krause-Sorio B. Siddarth P. Kilpatrick L. Milillo M. M. Aguilar-Faustino Y. Ercoli L.. (2022). Yoga prevents gray matter atrophy in women at risk for Alzheimer's disease: a randomized controlled trial. J. Alzheimers. Dis. 87, 569–581. 10.3233/JAD-215563 35275541 PMC9198760

[B58] Kurudenkandy F. R. Zilberter M. Biverstål H. Presto J. Honcharenko D. Strömberg R.. (2014). Amyloid-β-induced action potential desynchronization and degradation of hippocampal gamma oscillations is prevented by interference with peptide conformation change and aggregation. J. Neurosci. 34, 11416–11425. 10.1523/JNEUROSCI.1195-14.2014 25143621 PMC6615507

[B59] Kwok J. Y. Y. Kwan J. C. Y. Auyeung M. Mok V. C. T. Lau C. K. Y. Choi K. C.. (2019). Effects of mindfulness yoga vs stretching and resistance training exercises on anxiety and depression for people with Parkinson disease: a randomized clinical trial. JAMA Neurol. 76:755. 10.1001/jamaneurol.2019.0534 30958514 PMC6583059

[B60] Kwok J. Y. Y. Smith R. Chan L. M. L. Lam L. C. C. Fong D. Y. T. Choi E. P. H.. (2022). Managing freezing of gait in Parkinson's disease: a systematic review and network meta-analysis. J. Neurol. 269, 3310–3324. 10.1007/s00415-022-11031-z 35244766

[B61] Lee L.-H. N. Huang C.-S. Chuang H.-H. Lai H.-J. Yang C.-K. Yang Y.-C.. (2021). An electrophysiological perspective on Parkinson's disease: symptomatic pathogenesis and therapeutic approaches. J. Biomed. Sci. 28, 85. 10.1186/s12929-021-00781-z 34886870 PMC8656091

[B62] Lesinski M. Hortobágyi T. Muehlbauer T. Gollhofer A. Granacher U. (2015). Effects of balance training on balance performance in healthy older adults: a systematic review and meta-analysis. Sports Med. 45, 1721–1738. 10.1007/s40279-015-0375-y 26325622 PMC4656699

[B63] Liu X. (2021). Exercise improves movement by regulating the plasticity of cortical function in hemiparkinsonian rats. Front. Aging Neurosci. 13:695108. 10.3389/fnagi.2021.695108 34194319 PMC8236842

[B64] Liu Y. Yan T. Chu J. M.-T. Chen Y. Dunnett S. Ho Y.-S.. (2019). The beneficial effects of physical exercise in the brain and related pathophysiological mechanisms in neurodegenerative diseases. Labor. Invest. 99, 943–957. 10.1038/s41374-019-0232-y 30808929

[B65] López-Otín C. Kroemer G. (2021). Hallmarks of health. Cell 184, 33–63. 10.1016/j.cell.2020.11.034 33340459

[B66] Mably A. J. Colgin L. L. (2018). Gamma oscillations in cognitive disorders. Curr. Opin. Neurobiol. 52, 182–187. 10.1016/j.conb.2018.07.009 30121451 PMC6139067

[B67] Mably A. J. Gereke B. J. Jones D. T. Colgin L. L. (2017). Impairments in spatial representations and rhythmic coordination of place cells in the 3xTg mouse model of Alzheimer's disease. Hippocampus 27, 378–392. 10.1002/hipo.22697 28032686

[B68] Machado M. L. Lefèvre N. Philoxene B. Le Gall A. Madeleine S. Fleury P.. (2019). New software dedicated to virtual mazes for human cognitive investigations. J. Neurosci. Methods 327:108388. 10.1016/j.jneumeth.2019.108388 31408650

[B69] Mark J. I. Ryan H. Fabian K. DeMarco K. Lewek M. D. Cassidy J. M. (2023). Aerobic exercise and action observation priming modulate functional connectivity. PLoS ONE 18:e0283975. 10.1371/journal.pone.0283975 37023070 PMC10079047

[B70] Mattson M. P. Arumugam T. V. (2018). Hallmarks of brain aging: adaptive and pathological modification by metabolic states. Cell Metab. 27, 1176–1199. 10.1016/j.cmet.2018.05.011 29874566 PMC6039826

[B71] Mehak S. F. Shivakumar A. B. Kumari S. Muralidharan B. Gangadharan G. (2022). Theta and gamma oscillatory dynamics in mouse models of Alzheimer's disease: a path to prospective therapeutic intervention. Neurosci. Biobehav. Rev. 136:104628. 10.1016/j.neubiorev.2022.104628 35331816

[B72] Murphy M. H. Rowe D. A. Woods C. B. (2016). Sports participation in youth as a predictor of physical activity: a 5-year longitudinal study. J. Phys. Activity Health 13, 704–711. 10.1123/jpah.2015-0526 26800567

[B73] Murty D. V. P. S. Manikandan K. Santosh Kumar W. Garani Ramesh R. Purokayastha S. Javali M.. (2020). Gamma oscillations weaken with age in healthy elderly in human EEG. Neuroimage 215:116826. 10.1016/j.neuroimage.2020.116826 32276055 PMC7299665

[B74] Nicola L. Loo S. J. Q. Lyon G. Turknett J. Wood T. R. (2024). Does resistance training in older adults lead to structural brain changes associated with a lower risk of Alzheimer's dementia? A narrative review. Ageing Res. Rev. 98:102356. 10.1016/j.arr.2024.102356 38823487

[B75] Nobukawa S. Kikuchi M. Takahashi T. (2019). Changes in functional connectivity dynamics with aging: a dynamical phase synchronization approach. Neuroimage 188, 357–368. 10.1016/j.neuroimage.2018.12.008 30529509

[B76] Northey J. M. Cherbuin N. Pumpa K. L. Smee D. J. Rattray B. (2018). Exercise interventions for cognitive function in adults older than 50: a systematic review with meta-analysis. Br. J. Sports Med. 52, 154–160. 10.1136/bjsports-2016-096587 28438770

[B77] Nuzzo J. L. Pinto M. D. Kirk B. J. C. Nosaka K. (2024). Resistance exercise minimal dose strategies for increasing muscle strength in the general population: an overview. Sports Med. 54, 1139–1162. 10.1007/s40279-024-02009-0 38509414 PMC11127831

[B78] Ohia-Nwoko O. Montazari S. Lau Y. S. Eriksen J. L. (2014). Long-term treadmill exercise attenuates tau pathology in P301S tau transgenic mice. Mol. Neurodegener. 9, 1–17. 10.1186/1750-1326-9-54 25432085 PMC4280713

[B79] Oudbier S. J. Goh J. Looijaard S. M. L. M. Reijnierse E. M. Meskers C. G. M. Maier A. B. (2022). Pathophysiological mechanisms explaining the association between low skeletal muscle mass and cognitive function. J. Gerontol. A Biol. Sci. Med. Sci. 77, 1959–1968. 10.1093/gerona/glac121 35661882 PMC9536455

[B80] Palasz E. Niewiadomski W. Gasiorowska A. Mietelska-Porowska A. Niewiadomska G. (2019). Neuroplasticity and neuroprotective effect of treadmill training in the chronic mouse model of Parkinson's disease. Neural Plast. 2019, 1–14. 10.1155/2019/8215017 31073303 PMC6470436

[B81] Park S.-S. Park H.-S. Kim C.-J. Kang H.-S. Kim D.-H. Baek S.-S.. (2020). Physical exercise during exposure to 40-Hz light flicker improves cognitive functions in the 3xTg mouse model of Alzheimer's disease. Alzheimers. Res. Ther. 12:62. 10.1186/s13195-020-00631-4 32434556 PMC7240923

[B82] Pedroso R. V. Lima-Silva A. E. Tarachuque P. E. Fraga F. J. Stein A. M. (2021). Efficacy of physical exercise on cortical activity modulation in mild cognitive impairment: a systematic review. Arch. Phys. Med. Rehabil. 102, 2393–2401. 10.1016/j.apmr.2021.03.032 33932357

[B83] Peng Y. Jin H. Xue Y.-H. Chen Q. Yao S.-Y. Du M.-Q.. (2023). Current and future therapeutic strategies for Alzheimer's disease: an overview of drug development bottlenecks. Front. Aging Neurosci. 15:1206572. 10.3389/fnagi.2023.1206572 37600514 PMC10438465

[B84] Perinelli A. Assecondi S. Tagliabue C. F. Mazza V. (2022). Power shift and connectivity changes in healthy aging during resting-state EEG. Neuroimage 256:119247. 10.1016/j.neuroimage.2022.119247 35477019

[B85] Qi M. Zhu Y. Zhang L. Wu T. Wang J. (2019). The effect of aerobic dance intervention on brain spontaneous activity in older adults with mild cognitive impairment: a resting-state functional MRI study. Exp. Ther. Med. 17, 715–722. 10.3892/etm.2018.7006 30651855 PMC6307442

[B86] Qiu Y. Fernández-García B. Lehmann H. I. Li G. Kroemer G. López-Otín C.. (2023). Exercise sustains the hallmarks of health. J. Sport Health Sci. 12, 8–35. 10.1016/j.jshs.2022.10.003 36374766 PMC9923435

[B87] Rapp P. E. Keyser D. O. Albano A. Hernandez R. Gibson D. B. Zambon R. A.. (2015). Traumatic brain injury detection using electrophysiological methods. Front. Hum. Neurosci. 9:11. 10.3389/fnhum.2015.00011 25698950 PMC4316720

[B88] Rektorova I. Klobusiakova P. Balazova Z. Kropacova S. Sejnoha Minsterova A. Grmela R.. (2020). Brain structure changes in nondemented seniors after six-month dance-exercise intervention. Acta Neurol. Scand. 141, 90–97. 10.1111/ane.13181 31613387

[B89] Rosenblum Y. Shiner T. Bregman N. Fahoum F. Giladi N. Maidan I.. (2022). Event-related oscillations differentiate between cognitive, motor and visual impairments. J. Neurol. 269, 3529–3540. 10.1007/s00415-021-10953-4 35043223

[B90] Sáez de Asteasu M. L. Martínez-Velilla N. Zambom-Ferraresi F. Casas-Herrero Á. Izquierdo M. (2017). Role of physical exercise on cognitive function in healthy older adults: a systematic review of randomized clinical trials. Ageing Res. Rev. 37, 117–134. 10.1016/j.arr.2017.05.007 28587957

[B91] Schultz W. Stauffer W. R. Lak A. (2017). The phasic dopamine signal maturing: from reward via behavioural activation to formal economic utility. Curr. Opin. Neurobiol. 43, 139–148. 10.1016/j.conb.2017.03.013 28390863

[B92] Sebastián-Romagosa M. Cho W. Ortner R. Murovec N. Von Oertzen T. Kamada K.. (2020). Brain computer interface treatment for motor rehabilitation of upper extremity of stroke patients-a feasibility study. Front. Neurosci. 14:591435. 10.3389/fnins.2020.591435 33192277 PMC7640937

[B93] Sefat O. Salehinejad M. A. Danilewitz M. Shalbaf R. Vila-Rodriguez F. (2022). Combined yoga and transcranial direct current stimulation increase functional connectivity and synchronization in the frontal areas. Brain Topogr. 35, 207–218. 10.1007/s10548-022-00887-z 35092544

[B94] Steriade M. Gloor P. Llinás R. R. Lopes de Silva F. H. Mesulam M. M. (1990). Report of IFCN committee on basic mechanisms. Basic mechanisms of cerebral rhythmic activities. Electroencephalogr. Clin. Neurophysiol. 76, 481–508. 10.1016/0013-4694(90)90001-Z 1701118

[B95] Stern Y. MacKay-Brandt A. Lee S. McKinley P. McIntyre K. Razlighi Q.. (2019). Effect of aerobic exercise on cognition in younger adults. Neurology 92, e905–e916. 10.1212/WNL.0000000000007003 30700591 PMC6404470

[B96] Stone B. L. Beneda-Bender M. McCollum D. L. Sun J. Shelley J. H. Ashley J. D.. (2020). Understanding cognitive performance during exercise in Reserve Officers' Training Corps: establishing the executive function-exercise intensity relationship. J. Appl. Physiol. 129, 846–854. 10.1152/japplphysiol.00483.2020 32853115

[B97] Sudo M. Costello J. T. McMorris T. Ando S. (2022). The effects of acute high-intensity aerobic exercise on cognitive performance: a structured narrative review. Front. Behav. Neurosci. 16:957677. 10.3389/fnbeh.2022.957677 36212191 PMC9538359

[B98] Tapia-Rojas C. Aranguiz F. Varela-Nallar L. Inestrosa N. C. (2015). Voluntary running attenuates memory loss, decreases neuropathological changes and induces neurogenesis in a mouse model of Alzheimer's disease. Brain Pathol. 26, 62–74. 10.1111/bpa.12255 25763997 PMC8029165

[B99] Tavano A. Rimmele J. M. Michalareas G. Poeppel D. (2023). “Neural Oscillations in EEG and MEG,” in Language Electrified: Principles, Methods, and Future Perspectives of Investigation, eds. M. Grimaldi, E. Brattico, and Y. Shtyrov (New York, NY: Springer US), 241–284. 10.1007/978-1-0716-3263-5_8

[B100] Teel E. F. Ray W. J. Geronimo A. M. Slobounov S. M. (2014). Residual alterations of brain electrical activity in clinically asymptomatic concussed individuals: an EEG study. Clin. Neurophysiol. 125, 703–707. 10.1016/j.clinph.2013.08.027 24140103

[B101] Teixeira-Machado L. Arida R. M. de Jesus Mari J. (2019). Dance for neuroplasticity: a descriptive systematic review. Neurosci. Biobehav. Rev. 96, 232–240. 10.1016/j.neubiorev.2018.12.010 30543905

[B102] Tinkhauser G. Torrecillos F. Duclos Y. Tan H. Pogosyan A. Fischer P.. (2018). Beta burst coupling across the motor circuit in Parkinson's disease. Neurobiol. Dis. 117, 217–225. 10.1016/j.nbd.2018.06.007 29909050 PMC6054304

[B103] Tsai C.-L. Pai M.-C. Ukropec J. Ukropcová B. (2019). Distinctive effects of aerobic and resistance exercise modes on neurocognitive and biochemical changes in individuals with mild cognitive impairment. Curr. Alzheimer Res. 16, 316–332. 10.2174/1567205016666190228125429 30819077

[B104] Van Ede F. Quinn A. J. Woolrich M. W. Nobre A. C. (2018). Neural oscillations: sustained rhythms or transient burst-events? Trends Neurosci. 41, 415–417. 10.1016/j.tins.2018.04.004 29739627 PMC6024376

[B105] Vanderbeken I. Kerckhofs E. (2017). A systematic review of the effect of physical exercise on cognition in stroke and traumatic brain injury patients. NeuroRehabilitation 40, 33–48. 10.3233/NRE-161388 27814304

[B106] Vedovelli K. Giacobbo B. L. Corrêa M. S. Wieck A. Argimon I. I. de L.. (2017). Multimodal physical activity increases brain-derived neurotrophic factor levels and improves cognition in institutionalized older women. GeroScience 39, 407–417. 10.1007/s11357-017-9987-5 28707283 PMC5636777

[B107] Villemure C. Ceko M. Cotton V. A. Bushnell M. C. (2015). Neuroprotective effects of yoga practice: age-, experience-, and frequency-dependent plasticity. Front. Hum. Neurosci. 9:281. 10.3389/fnhum.2015.00281 26029093 PMC4428135

[B108] Vints W. A. J. Levin O. Fujiyama H. Verbunt J. Masiulis N. (2022). Exerkines and long-term synaptic potentiation: mechanisms of exercise-induced neuroplasticity. Front. Neuroendocrinol. 66:100993. 10.1016/j.yfrne.2022.100993 35283168

[B109] Voss J. L. Bridge D. J. Cohen N. J. Walker J. A. (2017a). A closer look at the hippocampus and memory. Trends Cogn. Sci. 21, 577–588. 10.1016/j.tics.2017.05.008 28625353 PMC5659202

[B110] Voss M. W. Vivar C. Kramer A. F. van Praag H. (2013). Bridging animal and human models of exercise-induced brain plasticity. Trends Cogn. Sci. 17, 525–544. 10.1016/j.tics.2013.08.001 24029446 PMC4565723

[B111] Voss P. Thomas M. E. Cisneros-Franco J. M. de Villers-Sidani É. (2017b). Dynamic brains and the changing rules of neuroplasticity: implications for learning and recovery. Front. Psychol. 8:1657. 10.3389/fpsyg.2017.01657 29085312 PMC5649212

[B112] Voss S. Cerna J. Gothe N. P. (2023). Yoga impacts cognitive health: neurophysiological changes and stress-regulation mechanisms. Exerc. Sport Sci. Rev. 51, 73–81. 10.1249/JES.0000000000000311 36342265 PMC10033324

[B113] Voytek B. Kramer M. A. Case J. Lepage K. Q. Tempesta Z. R. Knight R. T.. (2015). Age-related changes in 1/f neural electrophysiological noise. J. Neurosci. 35, 13257–13265. 10.1523/JNEUROSCI.2332-14.2015 26400953 PMC4579381

[B114] Wan X. Zhang Y. Liu T. Li D. Yu H. Wen D. (2024). Exercise therapy of mild cognitive impairment: EEG could enhance efficiency. Front. Aging Neurosci. 16. 10.3389/fnagi.2024.1373273 38659707 PMC11039927

[B115] Wang B. Li M. Haihambo N. Qiu Z. Sun M. Guo M.. (2024). Characterizing Major Depressive Disorder (MDD) using alpha-band activity in resting-state electroencephalogram (EEG) combined with MATRICS Consensus Cognitive Battery (MCCB). J. Affect. Disord. 355, 254–264. 10.1016/j.jad.2024.03.145 38561155

[B116] Wang J. Zhao X. Bi Y. Jiang S. Sun Y. Lang J.. (2023). Executive function elevated by long term high-intensity physical activity and the regulation role of beta-band activity in human frontal region. Cogn. Neurodyn. 17, 1463–1472. 10.1007/s11571-022-09905-z 37974584 PMC10640436

[B117] Wayne P. M. Walsh J. N. Taylor-Piliae R. E. Wells R. E. Papp K. V. Donovan N. J.. (2014). Effect of tai chi on cognitive performance in older adults: systematic review and meta-analysis. J. Am. Geriatr. Soc. 62, 25–39. 10.1111/jgs.12611 24383523 PMC4055508

[B118] Wei L. Chai Q. Chen J. Wang Q. Bao Y. Xu W.. (2022). The impact of Tai Chi on cognitive rehabilitation of elder adults with mild cognitive impairment: a systematic review and meta-analysis. Disabil. Rehabil. 44, 2197–2206. 10.1080/09638288.2020.1830311 33043709

[B119] Weiss E. Kann M. Wang Q. (2023). Neuromodulation of neural oscillations in health and disease. Biology 12:371. 10.3390/biology12030371 36979063 PMC10045166

[B120] Wheeler M. J. Green D. J. Ellis K. A. Cerin E. Heinonen I. Naylor L. H.. (2020). Distinct effects of acute exercise and breaks in sitting on working memory and executive function in older adults: a three-arm, randomised cross-over trial to evaluate the effects of exercise with and without breaks in sitting on cognition. Br. J. Sports Med. 54, 776–781. 10.1136/bjsports-2018-100168 31036563

[B121] Wu V. X. Chi Y. Lee J. K. Goh H. S. Chen D. Y. M. Haugan G.. (2021). The effect of dance interventions on cognition, neuroplasticity, physical function, depression, and quality of life for older adults with mild cognitive impairment: a systematic review and meta-analysis. Int. J. Nurs. Stud. 122:104025. 10.1016/j.ijnurstu.2021.104025 34298320

[B122] Yang G. Y. Hunter J. Bu F. L. Hao W. L. Zhang H. Wayne P. M.. (2022). Determining the safety and effectiveness of Tai Chi: a critical overview of 210 systematic reviews of controlled clinical trials. Syst. Rev. 11:260. 10.1186/s13643-022-02100-5 36463306 PMC9719113

[B123] Yang J. Zhang L. Tang Q. Wang F. Li Y. Peng H.. (2020). Tai chi is effective in delaying cognitive decline in older adults with mild cognitive impairment: evidence from a systematic review and meta-analysis. Evid. Based Complem. Altern. Med. 2020:3620534. 10.1155/2020/3620534 32308706 PMC7132349

[B124] Yu Y. Escobar Sanabria D. Wang J. Hendrix C. M. Zhang J. Nebeck S. D.. (2021). Parkinsonism alters beta burst dynamics across the basal ganglia–motor cortical network. J. Neurosci. 41, 2274–2286. 10.1523/JNEUROSCI.1591-20.2021 33483430 PMC8018776

[B125] Yue C. Yu Q. Zhang Y. Herold F. Mei J. Kong Z.. (2020). Regular tai chi practice is associated with improved memory as well as structural and functional alterations of the hippocampus in the elderly. Front. Aging Neurosci. 12:586770. 10.3389/fnagi.2020.586770 33192481 PMC7658399

[B126] Zhao J. Jiang W. Wang X. Cai Z. Liu Z. Liu G. (2020). Exercise, brain plasticity, and depression. CNS Neurosci. Ther. 26, 885–895. 10.1111/cns.13385 32491278 PMC7415205

[B127] Zheng K. Chen C. Yang S. Wang X. (2021). Aerobic exercise attenuates pain sensitivity: an event-related potential study. Front. Neurosci. 15:735470. 10.3389/fnins.2021.735470 34630022 PMC8494006

[B128] Zhu Y. Zhong Q. Ji J. Ma J. Wu H. Gao Y.. (2020). Effects of aerobic dance on cognition in older adults with mild cognitive impairment: a systematic review and meta-analysis. J. Alzheimer's Dis. 74, 679–690. 10.3233/JAD-190681 32083578

